# Hybrid peeling for fast and accurate calling, phasing, and imputation with sequence data of any coverage in pedigrees

**DOI:** 10.1186/s12711-018-0438-2

**Published:** 2018-12-18

**Authors:** Andrew Whalen, Roger Ros-Freixedes, David L. Wilson, Gregor Gorjanc, John M. Hickey

**Affiliations:** The Roslin Institute and Royal (Dick) School of Veterinary Studies, The University of Edinburgh, Midlothian, Scotland, UK

## Abstract

**Background:**

In this paper, we extend multi-locus iterative peeling to provide a computationally efficient method for calling, phasing, and imputing sequence data of any coverage in small or large pedigrees. Our method, called hybrid peeling, uses multi-locus iterative peeling to estimate shared chromosome segments between parents and their offspring at a subset of loci, and then uses single-locus iterative peeling to aggregate genomic information across multiple generations at the remaining loci.

**Results:**

Using a synthetic dataset, we first analysed the performance of hybrid peeling for calling and phasing genotypes in disconnected families, which contained only a focal individual and its parents and grandparents. Second, we analysed the performance of hybrid peeling for calling and phasing genotypes in the context of a full general pedigree. Third, we analysed the performance of hybrid peeling for imputing whole-genome sequence data to non-sequenced individuals in the population. We found that hybrid peeling substantially increased the number of called and phased genotypes by leveraging sequence information on related individuals. The calling rate and accuracy increased when the full pedigree was used compared to a reduced pedigree of just parents and grandparents. Finally, hybrid peeling imputed accurately whole-genome sequence to non-sequenced individuals.

**Conclusions:**

We believe that this algorithm will enable the generation of low cost and high accuracy whole-genome sequence data in many pedigreed populations. We make this algorithm available as a standalone program called AlphaPeel.

## Background

In this paper, we extend multi-locus iterative peeling to provide a computationally efficient method for calling, phasing, and imputing sequence data of any coverage in small or large pedigrees. In the past few years, the use of genomic data has expanded greatly. The widespread genotyping of animals empowers breeding via genomic selection [1, 2] and progress in biological knowledge via genome-wide association studies (GWAS) [3, 4]. The accuracy of genomic selection and the power of GWAS depend on both the number of individuals that have genomic data and the density of genomic data e.g., [5–8]. Thus, the goal is to generate genomic data on as many individuals as possible at the highest density possible with the upper limit being the presence of whole-genome sequence on hundreds of thousands or millions of individuals [9–11].

Although the cost of producing whole-genome sequence data for an individual has decreased substantially, it is still prohibitively expensive to obtain high coverage whole-genome sequence data on tens of thousands of individuals. An emerging strategy in breeding populations is to obtain a mix of high and low coverage sequence data on a subset of individuals, and then to propagate that information between related individuals to call whole-genome sequence genotypes for all members of a population, some of which may have only single nucleotide polymorphism (SNP) array genotype data [9]. This strategy exploits the high degree of relatedness and haplotype sharing between individuals in a breeding population, meaning that a haplotype can be inferred at high accuracy by low coverage sequencing of different individuals that share that haplotype. Algorithms for selecting the individuals to sequence in such a context have already been developed [12–14], but a method that could efficiently propagate the sequence information between related individuals is still lacking.

Past methods for using mixed coverage sequence data to call, phase, and impute genotypes have primarily exploited linkage disequilibrium (LD), e.g., MaCH [15], Beagle [16, 17]. LD-based methods perform well, particularly in human settings where individuals are mostly unrelated and there is limited pedigree data. However, these methods generally do not exploit explicit relationship information when pedigrees are available, but exceptions do exist [18, 19]. In contrast, pedigree-based methods can have a higher accuracy and lower computational cost than LD-based methods, particularly in populations with closely related individuals and accurate pedigrees across multiple generations e.g., [20–22]. Pedigree-based methods are particularly appealing for mixed coverage sequence data on relatives because they are able to collapse information across the long haplotype segments shared between individuals, their ancestors and their descendants.

Single-locus and multi-locus peeling are two pedigree-based methods that model an individual’s haplotypes based on the haplotypes of its parents and offspring and the individual’s genotype data. There is a large body of literature on peeling methods in genetics e.g., [21, 23–28] and related methods in other areas e.g., [29–31]. Since our interest is in efficient methods that could handle whole-genome sequence data in complex multi-generational pedigrees with loops, we focused on approximate (iterative) peeling methods, in particular on the single-locus method of Kerr and Kinghorn [32] and the multi-locus method of Meuwissen and Goddard [33]. In single-locus peeling, all loci are treated independently and so linkage between loci is not exploited. In contrast, multi-locus peeling tracks the linkage between loci allowing for information at one locus to be used at a neighbouring locus, which has a large potential with whole-genome sequence data. Although multi-locus peeling exploits more information and, thus, is more accurate, it is computationally more expensive due the high cost of calculating the segregation probabilities at each locus, and currently it is ill-suited for whole-genome sequence data.

In this paper, we present a hybrid peeling method that is scalable to whole-genome sequence data on tens of thousands of individuals. In hybrid peeling, segregation probabilities are calculated at a subset of loci (e.g., all of the loci on a high-density SNP array), and then fast single-locus style peeling operations are used at the remaining loci (e.g., the remaining segregating sites in whole-genome sequence). This approach exploits the benefits of using linkage from multi-locus peeling while still being able to scale to whole-genome sequence data on thousands of animals. In the following, we first present the hybrid peeling method, and then the results of its performance on a synthetic dataset based on a real commercial pig population with 65,000 animals on a single chromosome with 700,000 segregating loci.

## Methods

### Peeling methods

Peeling is a method for inferring the genotypes and underlying haplotypes of an individual based on their own genotype information and the genotype information of their ancestors and descendants. In this context, we refer to ‘calling an individual’s genotype’ to describe the process of inferring the genotype of an individual based on their own genetic information and the genetic information of their relatives. Furthermore, we use the term ‘phasing’ for determining the parent of origin for each allele at a heterozygous locus. Peeling is computationally intractable when considering whole-genome sequence in the context of large multi-generational pedigrees with loops [25, 28, 29, 34]. Iterative peeling approximates this problem through a series of peeling up and peeling down operations [32, 33, 35]. In the following, we refer to iterative peeling simply as peeling. In a peeling up operation, information from an individual’s descendants is used to infer the individual’s genotypes and allele origins. In a peeling down operation, information from an individual’s ancestors is used to infer the individual’s genotypes and allele origins. Repeating these operations propagates genetic information between members of a pedigree.

Peeling relies on a model of the transmission of genetic information between a parent and their offspring. Single-locus and multi-locus peeling differ in how they model this transmission. In single-locus peeling, both parental alleles are assumed to be inherited with equal probability at all loci. In multi-locus peeling, it is assumed that there is a high probability that the alleles at nearby loci are inherited jointly from the same parental haplotype. To share information between loci, multi-locus peeling estimates the segregation probabilities at each locus. These probabilities indicate which parental haplotypes were inherited at a given locus. Hybrid peeling is a computationally efficient approximation of multi-locus peeling. Like multi-locus peeling, it uses information from nearby loci to infer which parental haplotype was inherited at a locus. Unlike multi-locus peeling, it estimates segregation probabilities at a subset of loci only, and linearly interpolates segregation probabilities at the remaining loci.

For completeness, we describe these peeling operations in detail below. For single-locus peeling, we follow the previous work of Kerr and Kinghorn [32], and for multi-locus peeling, we follow the previous work of Meuwissen and Goddard [33].

### Single-locus peeling

Following Kerr and Kinghorn [32], in single-locus peeling we estimate the probability of an individual’s genotype at a locus as the product of three terms that represent the information from the genotype of their parents (anterior), the genotypes of their offspring (posterior), and their own genomic data (penetrance). For a biallelic locus, we have a set of four possible (phased) genotypes (*aa*, *aA*, *Aa*, *AA*), where the first allele in each pair is inherited from the father and the second allele is inherited from the mother. For brevity, we use the term phased genotype and genotype interchangeably. The probability that individual *i* has genotype g_i_ is:

1 $$p_{i} \left( {g_{i} } \right) \propto anterior_{i} \left( {g_{i} } \right)posterior_{i} \left( {g_{i} } \right)penetrance_{i} \left( {g_{i} } \right) .$$

We examine each of these terms separately.

The penetrance term gives the conditional probability of the available genomic data, i.e., SNP array data or sequencing data, given the individual’s genotype. If no information is available, we set the penetrance term to a constant value, i.e., $penetrance_{i} \left( {g_{i} } \right) = 1$. If we have SNP array data, we set $penetrance_{i} \left( {g_{i} } \right) = 1 - \varepsilon$ if *g*_*i*_ is consistent with the genotype called by the SNP array, and $penetrance_{i} \left( {g_{i} } \right) = \varepsilon$ otherwise, where *ε* accounts for a small error rate in SNP array genotype data. If we have sequencing data with $n_{ref}$ sequence reads of the reference allele, *a*, and $n_{alt}$ sequence reads of the alternative allele, *A*, then:

2 $$penetrance_{i} \left( {\begin{array}{*{20}l} {aa} \hfill \\ {aA} \hfill \\ {Aa} \hfill \\ {AA} \hfill \\ \end{array} } \right) \propto \left( {\begin{array}{*{20}c} {\left( {1 - \delta } \right)^{{n_{ref} }} \delta^{{n_{alt} }} } \\ {.5^{{n_{ref} + n_{alt} }} } \\ {.5^{{n_{ref} + n_{alt} }} } \\ {\delta^{{n_{ref} }} \left( {1 - \delta } \right)^{{n_{alt} }} } \\ \end{array} } \right) ,$$

where *δ* accounts for a small error rate in sequence data. By default, penetrance terms for an individual will not sum to 1, and so must be normalized to sum to 1.

The anterior probability captures the information about an individual’s genotype garnered from their parents’ genotypes. If the parents of the individual are unknown, then we use the minor allele frequency, *p*, to calculate the anterior probabilities:

3 $$anterior_{i} \left( {\begin{array}{*{20}l} {aa} \hfill \\ {aA} \hfill \\ {Aa} \hfill \\ {AA} \hfill \\ \end{array} } \right) \propto \left( {\begin{array}{*{20}c} {\left( {1 - p} \right)^{2} } \\ {p\left( {1 - p} \right)} \\ {p\left( {1 - p} \right)} \\ {p^{2} } \\ \end{array} } \right) .$$

When the parents of the individual are known, the anterior probabilities are:

4 $$anterior_{i} \left( {g_{i} } \right) = \mathop \sum \limits_{gm,gf} tr (g_{i} |g_{m} , g_{f} ) p_{ - i} \left( {g_{m} , g_{f} } \right) ,$$

where $p_{ - i} \left( {g_{m} , g_{f} } \right)$ is the joint probability that the mother has phased genotype *g*_*m*_ and the father has phased genotype *g*_*f*_ excluding any information from individual *i*. The “*tr*” term (a shorthand for “transmission”) is a function that gives the probability that the child inherits genotype *g*_*i*_ given their parent’s genotypes, i.e., $tr (g_{i} |g_{m} , g_{f} ) p_{ - i} \left( {g_{m} , g_{f} } \right)$. Examples of this function when inheriting from a single parent are in Table 1a. The joint probabilities of the parental genotypes are calculated by combining the anterior and posterior probabilities for both parents except for the information that pertains to individual *i*. This gives:

5 $$\begin{aligned} p_{ - i} \left( {g_{m} , g_{f} } \right) = &\,anterior_{m} \left( {g_{m} } \right)penetrance_{m} \left( {g_{m} } \right)posterior_{m, - f} \left( {g_{m} } \right), \\ & anterior_{f} \left( {g_{f} } \right)penetrance_{f} \left( {g_{f} } \right)posterior_{f, - m} \left( {g_{f} } \right) \\ & posterior_{f,m, - i} \left( {g_{m} ,g_{f} } \right), \\ \end{aligned}$$

where the value $p_{ - i} \left( {g_{m} , g_{f} } \right)$ is calculated as the product over the three lines. The first line calculates the probability of the mother’s genotype, *g*_*m*_, independent of shared children with *f*. The second line calculates the probability of the father’s genotype, *g*_*f*_, independent of shared children with *m*. The third line calculates the probability of both parents’ genotypes based on their shared children except for individual *i*.

**Table 1:** Examples of the transmission function under (a) single-locus peeling and (b) multi-locus peeling when the child inherits the grandpaternal (first) allele

(a) Single-locus peeling			(b) Multi-locus peeling		
Paternal genotype	Inherited allele	Probability	Paternal genotype	Inherited allele	Probability
aa	a	1	aa	a	1
aA	a	0.5	aA	a	1
Aa	a	0.5	Aa	a	0
AA	a	0	AA	a	0
aa	A	0	aa	A	0
aA	A	0.5	aA	A	0
Aa	A	0.5	Aa	A	1
AA	A	1	AA	A	1

There are two types of posterior terms. First, $posterior_{f,m}$ is the joint probability of the parents’ genotypes, based on all their shared children. Second, $posterior_{m}$ is the probability of a single parent’s genotype based on all of its mates and children. We can calculate $posterior_{f,m}$ by:

6a $$posterior_{m,f} \left( {g_{m} , g_{f} } \right) = \mathop \prod \limits_{c} \mathop \sum \limits_{{g_{c} }} tr\left( {g_{c} |g_{m} ,g_{f} } \right)posterior_{c} \left( {g_{c} } \right)penetrance_{c} \left( {g_{c} } \right) ,$$

which is the product of the probability that a child, *c*, inherits genotype *g*_*c*_, based on its parent’s genotypes, marginalized over the genotypes for *c*, and multiplied across all children. We then calculate $posterior_{m} \left( {g_{m} } \right)$ as the product of the $posterior_{m,f} \left( {g_{m} , g_{f} } \right)$ for all of the mates of *m* marginalized over their possible genotypes:

6b $$posterior_{m} \left( {g_{m} } \right) = \mathop \prod \limits_{k} \mathop \sum \limits_{{g_{k} }} posterior_{m,k} \left( {g_{m} , g_{k} } \right)p\left( {g_{m} ,g_{k} } \right) .$$

The remaining terms are calculated by removing the individuals that relate to them in the equations:

6c $$posterior_{m,f, - i} \left( {g_{m} , g_{f} } \right) = \mathop \prod \limits_{c \ne i} \mathop \sum \limits_{{g_{c} }} tr(g_{c} | g_{m} , g_{f} )posterior_{c} \left( {g_{c} } \right)penetrance_{c} \left( {g_{c} } \right) .$$

Together the posterior, anterior, and penetrance terms give the probability of an individual’s genotypes (Eq. 1). Information from the individual’s siblings and parents is contained in the anterior term. Information from an individual’s children and mates is contained in the posterior term. Information from more distant relatives is included via anterior and posterior terms of the individual’s immediate relatives. An individual’s own genetic information is contained in the penetrance function. When estimating the genotype probabilities for a set of parents in the anterior term, the focal individual’s penetrance and anterior terms are excluded from the calculation (Eq. 5), which ensures that information from an individual is included only in the anterior or posterior term but not both.

Due to the dependence of the anterior terms and posterior terms on the anterior and posterior terms of other individuals in the population, the order in which they are updated is important and can decrease the overall number of peeling operations that need to be performed. We follow the updating pattern given in [32]. At the start, we initialize all the posterior terms to a constant value, i.e., 1. Next, we peel down the pedigree from the oldest to the youngest generation, updating the anterior terms and then peel up the pedigree, updating the posterior terms. These peeling operations are repeated until the genotype probabilities for all of the individuals in the population converge, or for a maximum of 20 cycles of peeling.

There are two model parameters that need to be estimated, the minor allele frequency, *p*, and error rates, *ε* and *δ*. We found that an easy way to update these is by setting them equal to their observed values after each pair of peeling (up and down) operations. For the minor allele frequency, *p*, we average allele dosages for all the founders in the population (i.e., individuals without known ancestors):

7 $${\text{p}} = \frac{ 1}{\text{n}}\mathop \sum \limits_{\text{i}} p_{i} \left( {aA} \right) + p_{i} \left( {Aa} \right) + 2p_{i} \left( {AA} \right) ,$$

where the sum is over each of the *n* founders and $p_{i} \left( g \right)$ gives the probability that individual *i* has genotype *g* based on the current estimates (Eq. 1). For the error rate, we calculate the proportion of errors at each locus, i.e., the probability that an individual’s true genotype and observed genotype do not match.

For array data, we calculate:

8 $$\varepsilon = \frac{1}{n}\mathop \sum \limits_{i} \mathop \sum \limits_{{g_{i} }} I\left( {d_{i} \ne g_{i} } \right)p\left( {g_{i} } \right) ,$$

where $I\left( {d_{i} \ne g_{i} } \right)$ is an indicator function which is 1 if the observed genotype (a value of 0, 1, or 2) disagrees with the estimated underlying genotype, and 0 if they agree.

For sequence data, we estimated the error rate based on the number of incorrect reads found for homozygous loci, ignoring heterozygous loci:

9 $$\delta = \frac{{\mathop \sum \nolimits_{i} n_{i, alt} p_{i} \left( {aa} \right) + n_{i,ref} p_{i} \left( {AA} \right)}}{{\mathop \sum \nolimits_{i} \left( {n_{i,ref} + n_{i,alt} } \right)\left( {p_{i} \left( {aa} \right) + p_{i} \left( {AA} \right)} \right)}} .$$

In the numerator, we estimate the number of errors for a given individual. The term $n_{i, alt} p_{i} \left( {aa} \right)$ gives the number of reads for the alternative allele for individual *i*, times the probability that individual *i* is homozygous for the reference allele. The denominator is the total number of reads on a given individual multiplied by the probability that the individual is either homozygous for the reference allele or homozygous for the alternative allele. We tested either a single error rate for all loci or a locus-specific error rate and found that the locus-specific error rate leads to a slight increase in accuracy and, thus, we used a locus-specific error rate for *ε* and *δ*.

### Multi-locus peeling

Multi-locus peeling extends single-locus peeling by modifying the transmission function such that it is sensitive to which parental haplotype was inherited at a locus. In single-locus peeling, we assume that each parental haplotype is inherited with equal probability, and that the alleles at neighbouring loci are inherited independently, but this is not the case due to the small number of recombinations per chromosome causing children to inherit parental haplotypes in large blocks. This means that if we can identify which parental haplotype was inherited at one locus, we also know which haplotype was likely inherited at nearby loci. In the context of the peeling operations, if we know which haplotype was inherited, we can modify the peeling operations so that only the alleles from that haplotype will be transmitted, as demonstrated in Table 1b. Uncertainty about which haplotype is inherited can be incorporated in the model by marginalizing over possible inherited haplotypes.

More formally, we track the set of inherited haplotypes in terms of a segregation probability, which gives the probability that a child inherits one of the four possible pairs of parental haplotypes, $s \in \left\{ {pp, pm, mp, mm} \right\}$; relating to whether the father (first allele) or the mother (second allele) transmits their paternal (*p*) or maternal (*m*) haplotype at a locus. We can then build the transmission function by marginalizing over segregation states:

10 $$tr (g_{i} |g_{m} , g_{f} ) = \mathop \sum \limits_{s} tr (g_{i} |g_{m} , g_{f} , seg_{i,j} = s) p\left( {seg_{i,j} = s} \right) ,$$

where $p\left( {seg_{i,j} = s} \right)$ is the probability that individual *i* has segregation *s* at locus *j*. $tr (g_{i} |g_{m} , g_{f} , seg_{i,j} = s)$ is the probability that the child inherits genotype *g*_*i*_ given its paternal genotypes and their segregation (see Table 1b for an example). To perform peeling, we substitute the transmission function in Eqs. 4, 6a, and 6c with the transmission function in Eq. 10.

The segregation probabilities at each locus are calculated via the forward–backward algorithm [36]. In the algorithm, we calculate the segregation probabilities from three terms:

11a $$\begin{aligned} p\left( {seg_{{i,j}} = s} \right) = &~p\left( {seg_{{i,j}} = s,~seg_{{i,j - 1}} } \right) \\ &~p~(seg_{{i,j}} = s,~g_{i} ,~g_{f} ,~g_{m} ) \\&~p\left( {seg_{{i,j}} = s,seg_{{i,j + 1}} } \right), \\ \end{aligned}$$

where e.g.,

11b $$\begin{aligned} & p\left( {seg_{i,j} = s, seg_{i,j - 1} } \right) \\ & = \mathop \sum \limits_{{s^{{\prime }} }} p\left( {seg_{i,j} = s| seg_{i,j - 1} = s^{{\prime }} } \right)p\left( {seg_{i,j - 1} = s^{{\prime }} ,seg_{i,j - 2} } \right).\\ \end{aligned}$$

In Eq. (11a), the first and the last terms, $p\left( {seg_{i,j} = s, seg_{i,j - 1} } \right)$ and $p\left( {seg_{i,j} = s,seg_{i,j + 1} } \right)$, provide information about the segregation probabilities at locus *j* based on the segregation at the locus immediately before, and immediately after. In Eq. (11b), we explicitly marginalize over the segregation probabilities at loci *j* − 1 (or *j* + 1), using information only from the markers before (or after) *j*. We set the loci-to-loci transmission function to:

$$p\left( {seg_{i,j} = s |seg_{i,j - 1} = s^{{\prime }} } \right) = \left( {1 - \gamma } \right)^{2 - nChanges} \gamma^{nChanges},$$

where $nChanges$ is the number of differences in the segregation (up to 2) between *s* and $s^{{\prime }}$, and *γ* is recombination rate, i.e., $p\left( {seg_{i,j} = pp |seg_{i,j - 1} = pm} \right) = \left( {1 - \gamma } \right)\gamma$. The calculation of segregation probabilities differs from that of Meuwissen and Goddard [33], since here, we infer the segregation probabilities for both parents simultaneously, whereas Meuwissen and Goddard [33] infer the segregation probabilities for each parent separately (and assume that the probabilities are independent of each other).

The middle term, $p\left( {seg_{i,j} ,g_{i} , g_{f} ,g_{m} } \right)$, gives estimates for the segregation probabilities based on the information at locus *j*, as follows:

12 $$\begin{aligned} p\left( {seg_{i,j} ,g_{i} , g_{f} ,g_{m} } \right) & = tr\left( {g_{i} |g_{f} ,g_{m} ,seg_{i,j} } \right)penetrance_{i} \left( {g_{i} } \right)posterior_{i} \left( {g_{i} } \right) \\ & \quad anterior_{m} \left( {g_{m} } \right)penetrance_{m} \left( {g_{m} } \right)posterior_{m, - f} \left( {g_{m} } \right) \\ & \quad anterior_{f} \left( {g_{f} } \right)penetrance_{f} \left( {g_{f} } \right)posterior_{f, - m} \left( {g_{f} } \right) \\ & \quad posterior_{m,f, - i} \left( {g_{m} , g_{f} } \right). \\ \end{aligned}$$

The first line is the probability of the individual’s genotype, the second is the probability of the mother’s genotype, the third is the probability of the father’s genotype, and the fourth is the joint probability of the parents’ genotype. It is worth noting that none of the posterior or anterior terms in Eq. (12) rely on $tr\left( {g_{i} |g_{f} ,g_{m} ,seg_{i,j} } \right)$. The transmission term for an individual governs which alleles an individual is likely to have inherited from their parents, and is a key part of the anterior term for the focal individual, $anterior_{i} \left( {g_{i} } \right)$ and the posterior term for their parents, $posterior_{m,f} \left( {g_{m} , g_{f} } \right)$. The anterior term is excluded from Eq. (12), as is the contribution of individual *i* to the joint posterior term of their parents.

Multi-locus peeling is performed as a combination of forward–backward operations over loci and up-down operations over individuals. Forward–backward operations update segregation probabilities between loci for each individual, while up-down operations update anterior and posterior terms and genotype probabilities for each individual. At the end of each pass, we update the minor allele frequency, *p*, error rates, *ε* and *δ*, and recombination rate, *γ.* The minor allele frequency and error rates are updated as in single-locus peeling. The recombination rate is updated by estimating the number of recombinations that occurred between loci by examining the difference between the segregation estimates at adjacent loci:

13 $$\begin{aligned} \gamma & = \frac{1}{n}\mathop \sum \limits_{i} \mathop \sum \limits_{{seg_{i,j} }} \mathop \sum \limits_{{seg_{i,j + 1} }} I\left( {seg_{i,j} \ne seg_{i,j + 1} } \right) \\ & \quad \times p\left( {seg_{i,j} |seg_{i,j - 1} } \right)p\left( {seg_{i,j} , g_{i} ,g_{f} ,g_{m} } \right)p(seg_{i,j + 1} |seg_{i,j + 2} ). \\ \end{aligned}$$

Similar to the error rate, we found that using a locus-specific recombination rate slightly increased accuracy, and thus, we used a locus-specific recombination rate. Pilot simulations found that the genotype probabilities converged at around 10 to 20 cycles in large multi-generational livestock pedigrees with 60,000+ members, and thus we ran the algorithm for a fixed number of 20 cycles.

### Hybrid peeling

Hybrid peeling is a computationally efficient approximation to multi-locus peeling. In a preliminary analysis, we found that the primary computational cost of multi-locus peeling stemmed from updating the segregation probabilities, Eq. (13). When evaluating many loci on a chromosome, we expect that the segregation probabilities at nearby loci to be nearly equal. Because of this, it should be possible to evaluate the segregation probabilities at a subset of loci, and interpolate segregation probabilities at the remaining loci. Then, these estimates can be used to create a new transmission function for peeling operations.

More formally, we divided the set of loci into two sets, A and B, with the size of A being much smaller than B, |A| ≪ |B|, e.g., A are the loci on a high-density SNP array and B is the entire set of segregating loci obtained from whole-genome sequencing. We performed multi-locus peeling on the loci in A to calculate segregation probabilities and then single-locus peeling on the loci in B using Eq. (10) as the transmission function with interpolated segregation probabilities:

14 $$p\left( {seg_{i,k} = s} \right) = a p\left( {seg_{i,j} = s} \right) + \left( {1 - a} \right)p\left( {seg_{i,j + 1} = s} \right) ,$$

where *j* and *j* + 1 are the loci in the set A that flank locus *k*, and *a* is the proportional distance between locus *k* and locus *j*:

15 $$a = \frac{{d\left( {k,j} \right)}}{{d\left( {j, j + 1} \right)}} .$$

The distance can be calculated in number of base pairs, centiMorgans, or number of intermediary loci. The exact measure of distance should only have a minimal impact on performance: if a sufficiently large number of loci is used in set A then adjacent segregation probabilities should be nearly equal, i.e., $p\left( {seg_{i,j} = s} \right) = p\left( {seg_{i,j + 1} = s} \right)$ leading Eq. (14) to reduce to $p\left( {seg_{i,j} = s} \right)$ and no longer depend on the distance metric used.

The aim of the hybrid technique is to make multi-locus peeling more computationally tractable when applying it to large pedigrees. We evaluated the performance of this algorithm on a synthetic dataset.

### Analysis

We examined the performance of hybrid peeling for calling, phasing, and imputing sequence data of different coverages in pedigrees. To perform these analyses, we simulated genomes for 65,000 animals using a multi-generational pedigree derived from a real commercial pig breeding line. We assumed that some animals had high-density or low-density SNP array genotypes from routine genomic selection. In addition, we generated mixed coverage sequence data for a subset of focal animals. Then, we carried out three sets of analyses. First, we analysed the performance of hybrid peeling for calling and phasing in disconnected families, these are subsets of the pedigree, which contained only a focal animal and its parents and grandparents. Second, we analysed the performance of hybrid peeling for calling and phasing in the context of the full general pedigree. Third, we analysed the performance of hybrid peeling for whole-genome sequence imputation. In the following, we describe in detail how we simulated and analysed the data.

### Data

Genomes were generated using the Markovian Coalescent Simulator (MaCS) [37] and AlphaSim [38]. We generated 1000 base haplotypes for each of 10 chromosomes, assuming a chromosome length of 10^8^ base pairs, a per site mutation rate of 2.5 × 10^−8^, a between-site recombination rate of 1 × 10^−8^, and an effective population size (*N*_*e*_) that varied over time in accordance with estimates for a livestock population [39]. The resulting haplotypes had about 700,000 segregating loci per chromosome. On each of the chromosomes, we designated 2000 evenly distributed loci as markers on a high-density SNP array and a subset of 500 as markers on a low-density SNP array.

We used AlphaSim to drop the base haplotypes through a multi-generational pedigree of 65,000 animals from a real commercial pig breeding line. We assigned SNP array data to animals, in line with routine genotyping for genomic selection in the population; 45,000 animals were genotyped with the high-density SNP array, 10,000 animals were genotyped with the low-density SNP array, and 10,000 animals were not genotyped.

We generated sequence data, in line with the strategies implemented in the population. The goal was to use roughly $300,000 worth of sequencing resources to sequence and impute the entire population. First, the top 475 sires (all sires with more than 25 progeny) were sequenced at 2x coverage. Second, AlphaSeqOpt [13] was used to identify focal animals and their parents and grandparents (211 in total) to be sequenced and the coverages that they should be sequenced at. In AlphaSeqOpt, focal individuals are individuals who carry the most frequent haplotypes in the population. Once focal individuals are selected, then AlphaSeqOpt distributes a fixed sequencing budget among the focal individuals and their relatives to maximise the proportion of haplotypes in the population that are sequenced and could be subsequently phased with high accuracy using heuristics. AlphaSeqOpt was run using the high-density SNP array data on all chromosomes with an option to assign an individual sequencing coverage of either 1x, 2x, 15x, or 30x, and a total budget of $71,000. Third, the top 50 dams (based on the number of progeny and grand-progeny with and without a sequenced sire) were sequenced at 2x and the next 450 dams were sequenced at 1x. Finally, AlphaSeqOpt2 [14] was used to identify 800 individuals to be sequenced at 1x, to top-up the accumulated coverage of common haplotypes to 10x.

In total, we generated sequenced data for 1912 animals at a range of coverages for a cost of $289,312. We partitioned this data into three sets: (1) the *focal* identified with AlphaSeqOpt, (2) the *focal plus low coverage sires* which also included the top 475 sires, and (3) *focal plus all low coverage individuals*, i.e. all the sequenced animals. A breakdown of the total cost and sequencing coverage by these sets is in Table 2. We assumed that the cost of obtaining a DNA library for an individual was $39 and the cost of sequencing that library for an individual at 1x was $68, 2x was $136, 15x was $408, and 30x was $816. The costs were assumed to be non-linear to reflect current industry costs.

**Table 2:** Number of sequenced animals and cost by sequence coverage for the three sequencing sets

Coverage	Focal		Focal and low coverage sires		Focal and all low coverage	
	N	Cost ($)	N	Cost ($)	N	Cost ($)
1x	33	3531	33	3531	1282	137,174
2x	78	13,650	479	83,825	530	92,750
15x	64	28,608	64	28,608	64	28,608
30x	36	30,780	36	30,780	36	30,780
Total	211	76,569	612	146,744	1912	289,312

Sequence data were simulated by sampling sequencing reads for the 700,000 segregating loci on chromosome 10. The number of reads was generated using a Poisson-Gamma distribution, which allowed the number of sequence reads per locus to vary along the genome and between individuals [15]. First, a sequenceability (*γ*_*j*_) of each of the 700,000 loci along the genome was sampled from a gamma distribution, with shape and scale parameters respectively equal to $\alpha = 4$ and $1/\alpha = 0.25$. Second, the number of reads (*γ*_*i,j*_) per individual *i* at locus *j* was sampled from a Poisson distribution with a mean equal to $\mu_{i,j} = x_{i} y_{j}$, where *x*_*i*_ was the targeted coverage for individual *i*. Third, sequencing reads were generated by randomly sampling alleles from the two gametes of individual *i* at locus *j*, accounting for a sequencing error ($\varepsilon = 0.001$).

### Calling and phasing in disconnected families

We tested the ability of hybrid peeling to call genotypes and phase alleles in sequenced individuals using information from their parents and grandparents. For this, we selected 10 disconnected families (consisting of a focal individual and its parents and grandparents) from the full pedigree, and analysed the effect of sequencing coverage on our ability to call and phase the individual’s genotypes. To perform this, we ran the hybrid peeling when the focal individual was sequenced at 1x, 2x, 5x, 15x, or 30x coverage, and when its parents or grandparents were sequenced at 0x, 1x, 2x, 5x, 15x, or 30x coverage. We generated data for each of these scenarios separately. We assumed that all the parents or all the grandparents were sequenced at the same coverage, and that all family members had high-density SNP array data.

To call genotypes and phase alleles, we extracted the phased genotype probabilities generated by hybrid peeling and made a call if the probability of a genotype was higher than a pre-defined threshold. For all analyses, we used a probability threshold of 0.98. Scenarios were compared on the percentage of called genotypes (genotype yield) and phased alleles (phase yield).

### Calling and phasing with the full pedigree

Next, we tested the ability of hybrid peeling to call genotypes and phase alleles in sequenced individuals using information from the full pedigree. To perform this, we ran hybrid peeling twice. First, we ran it separately for each disconnected family that consisted of an individual, its parents, and grandparents, with (potentially missing or low coverage) SNP array and sequence data. Second, we ran it with SNP array and sequence data on all individuals in the pedigree. The sequencing coverage for each individual was the same as its coverage in the *focal and all low coverage* condition (see the Data subsection). We compared the genotype and phase yield between runs and compared the correlation between individual’s genotype dosages and the true genotypes (genotype accuracy) and correlation between individual’s allele dosages and the true alleles (phase accuracy) between runs.

### Imputing whole-genome sequence

Last, we tested the ability of hybrid peeling to impute whole-genome sequence for non-sequenced individuals in the full pedigree. We ran hybrid peeling on all the individuals in the full pedigree. The multi-locus step used the high-density SNP array loci to estimate segregation probabilities. Then, hybrid peeling was run on the sequence data using all available sequence and SNP array data. Hybrid peeling was run three times, using either the sequence data from the *focal*, *focal and low coverage sires*, or *focal and all low coverage* conditions.

Imputation accuracy was measured as the correlation between an individual’s imputed genotype dosages and the true genotypes. This measure of imputation accuracy has been reported to give imputation accuracies that are higher than the resulting genomic prediction accuracy [40]. Correcting for allele frequency is often recommended to improve the relationship between imputation accuracy and genomic prediction accuracy, particularly in the context of population-based imputation algorithms (e.g., [17]) where the default prediction for an individual’s allele is the major allele. The advantage of correcting for allele frequency in the context of pedigree-based imputation is less clear and may result in imputation accuracies that are still inflated. This is compounded by the fact that allele frequency is not clearly defined for a structured population, and by itself has limited impact on the imputed genotypes of a specific individual (which depends primarily on the frequency of the allele in the parents and other close relatives). Because of this, we did not correct allele frequency in this study, but we wish to highlight the fact that the imputation accuracies obtained here may not directly reflect the value of the genetic data for downstream analyses (e.g., genomic best linear unbiased prediction (GBLUP) or GWAS).

To provide a comparison to hybrid peeling, we also tested findhap v4 [22], a combined pedigree and LD-based imputation algorithm that can impute low to medium coverage sequence data. We ran findhap using a maximum segment length of 10,000, minimum segment length of 1000, and sequencing error rate of 0.01.

### Data availability

Simulated genotype and sequence data are available from the authors upon request.

### Code availability

To perform hybrid peeling, we used the software package AlphaPeel, which is available from the AlphaGenes website (http://www.alphagenes.roslin.ed.ac.uk). The code for generating simulated sequence data from genotype data is available from the authors on request.

## Results

Overall, we found that hybrid peeling had a high yield and accuracy for calling genotypes and phasing alleles. It also had a high accuracy for imputing whole-genome sequence data to non-sequenced individuals.

### Calling and phasing in disconnected families

**Fig. 1:**
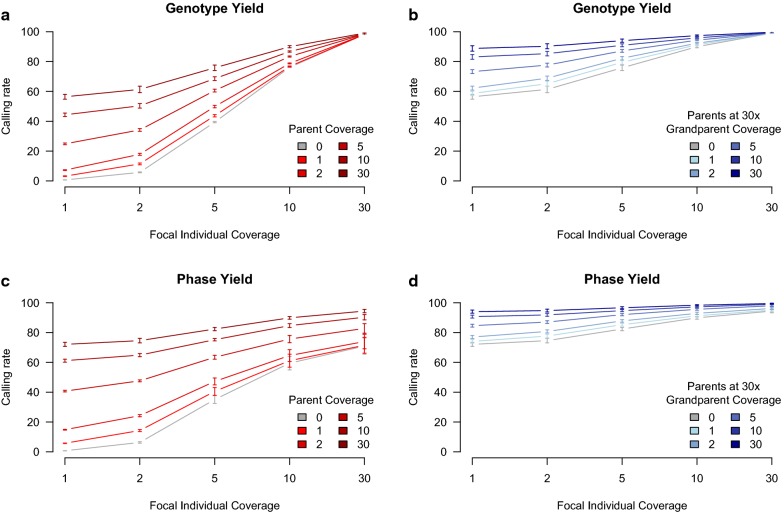
Genotype and phase yield with varying sequence coverage of the focal individual and its parents and grandparents. **a**, **b** Give the percentage of called genotypes when varying **a** the sequence coverage of parents and **b** grandparents. **c**, **d** Give the percentage of phased alleles when varying **c** the sequence coverage of parents and **d** grandparents. In **a**, **c** the sequence coverage of grandparents was 0x. In **b**, **d** the sequence coverage of parents was kept constant at 30x. In all four panels, the accuracy of calling genotypes and phasing alleles was higher than 0.98. Error bars represent plus and minus one standard error based on ten replications

We found that hybrid peeling yielded a high percentage and accuracy of called genotypes and phased alleles even with low coverage sequencing. The yields in each simulation are in Fig. 1.

The primary determinant for the percentage of called genotypes was the individual’s own degree of sequencing coverage. If neither the parents nor the grandparents of an individual were sequenced, and if the individual was sequenced at 1x, the percentage of called genotypes was 0.6%, and increased to 5% at 2x, 39% at 5x, 76% at 10x, and 98% at 30x. These values greatly increased if the parents were sequenced at high coverage. If the individual’s parents were both sequenced at 30x, then the percentage of called genotypes was 56% at 1x, 61% at 2, 75% at 5x, 90% at 10x, and 99% at 30x. Adding additional coverage on grandparents increased the accuracy of called genotypes even if the parents had 30x coverage. If both the parents and the grandparents had 30x coverage then the percentage of called genotypes reached 88% at 1x, 90% at 2x, 94% at 5x, 97% at 10x, and 99% at 30x. In all cases, the percentage of correctly called genotypes was higher than 0.995 (median 0.999).

We obtained a similar pattern of results when evaluating phasing yield. In this case, although an individual’s own sequencing coverage was an important determinant for phasing yield, high coverage on both the parents and the grandparents was needed to phase the alleles. If neither the parents nor the grandparents of an individual were sequenced, then phasing yield was 0.7% at 1x, 6% at 2x, 35% at 5x, 59% at 10x, and 70% at 30x. The low phasing yield at 30x is due to the inability to phase heterozygous loci without information from relatives. Sequencing the parents at high coverage substantially increased phasing yield, and continued to do so even if the individual was sequenced at high coverage. If the parents of the individual were sequenced at 30x, then phasing yield was 72% at 1x, 74% at 2x, 82% at 5x, 89% at 10x and 94% at 30x. If both the individual’s parents and grandparents were sequenced at 30x, then phasing yield increased to 94% at 1x, 95% at 2x, 96% at 5x, 98% at 10x, and 99% and 30x. In all cases, the percentage of correctly phased alleles was higher than 0.989 (median 0.999).

### Calling and phasing with the full pedigree

**Fig. 2:**
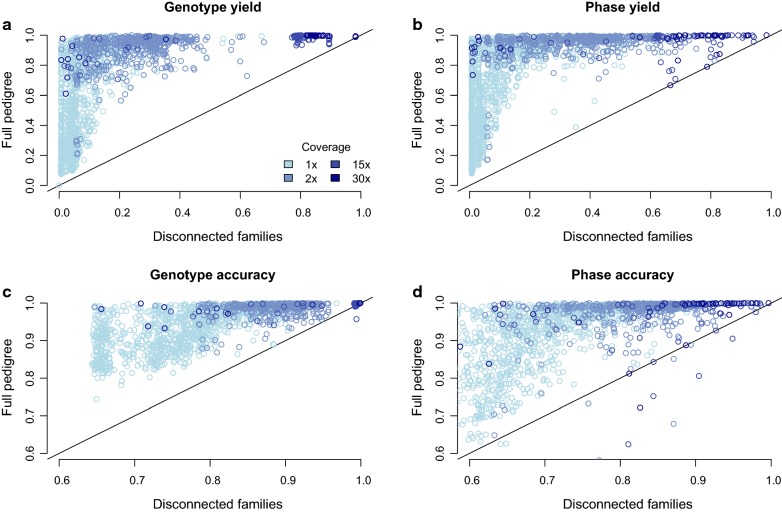
Genotype, and phase yield and accuracy when hybrid peeling is run on a series of disconnected families containing a focal individual and its parents and grandparents, or as part of the full general pedigree. **a**, **c** Compare the performance of calling genotypes, measured either with **a** the genotype yield or **c** the correlation between individual’s genotype dosages and the true genotypes (accuracy). **b**, **d** Compare the performance of phasing alleles, measured either with **b** the phase yield, or **d** the correlation between an individual’s allele dosages and the true alleles (accuracy)

We examined the effect of using all sequence data from the full pedigree on percentage of called genotypes and phasing yield and accuracy of sequenced individuals. The gains in yield and accuracy in comparison to using data from disconnected families are plotted in Fig. 2. We found that including the full pedigree greatly increased both percentage of called genotypes and phasing yield and accuracy. The gains were smaller for high coverage individuals compared to low coverage individuals. For example, phase accuracy increased on average from 0.85 to 0.97 for 30x individuals, but increased on average from 0.67 to 0.89 for 1x individuals.

The gains in phasing accuracy were also not equal for all individuals in the pedigree; some individuals had only a small gain in accuracy, whereas others had a large gain in accuracy. This difference was particularly pronounced for individuals sequenced at 1x where the mean phasing yield increased from 0.11 to 0.67, but the standard deviation of the phasing yield increased from 0.13 to 0.28. If all individuals were influenced equally by including the full pedigree, we should expect an increase in mean but not a corresponding increase in standard deviation. The increased variability is a consequence of the different sequencing coverages on relatives who are outside of the immediate family. We found that the amount of sequencing coverage on immediate relatives (parents and grandparents) is a good predictor of the phase accuracy of individuals sequenced at 1x in the disconnected family (Pearson correlation r^2^ = 0.37), but is a weak predictor for the phase accuracy of those individuals in the full pedigree (r^2^ = 0.13). In contrast, adding the sequencing coverage on all ancestors increased the ability to predict accuracy when assessing the phase accuracy in the full pedigree (r^2^ increased from 0.13 to 0.42), compared to when assessing the phase accuracy in the disconnected families, (r^2^ increased from 0.37 to 0.55). The higher overall r^2^ for disconnected families is likely due to performance in a disconnected family being easier to estimate because of the limited interaction between coverage levels for distant ancestors. We found a similar pattern of results for genotype accuracy and the percentage of called genotypes and phasing yield.

### Imputing whole-genome sequence

**Fig. 3:**
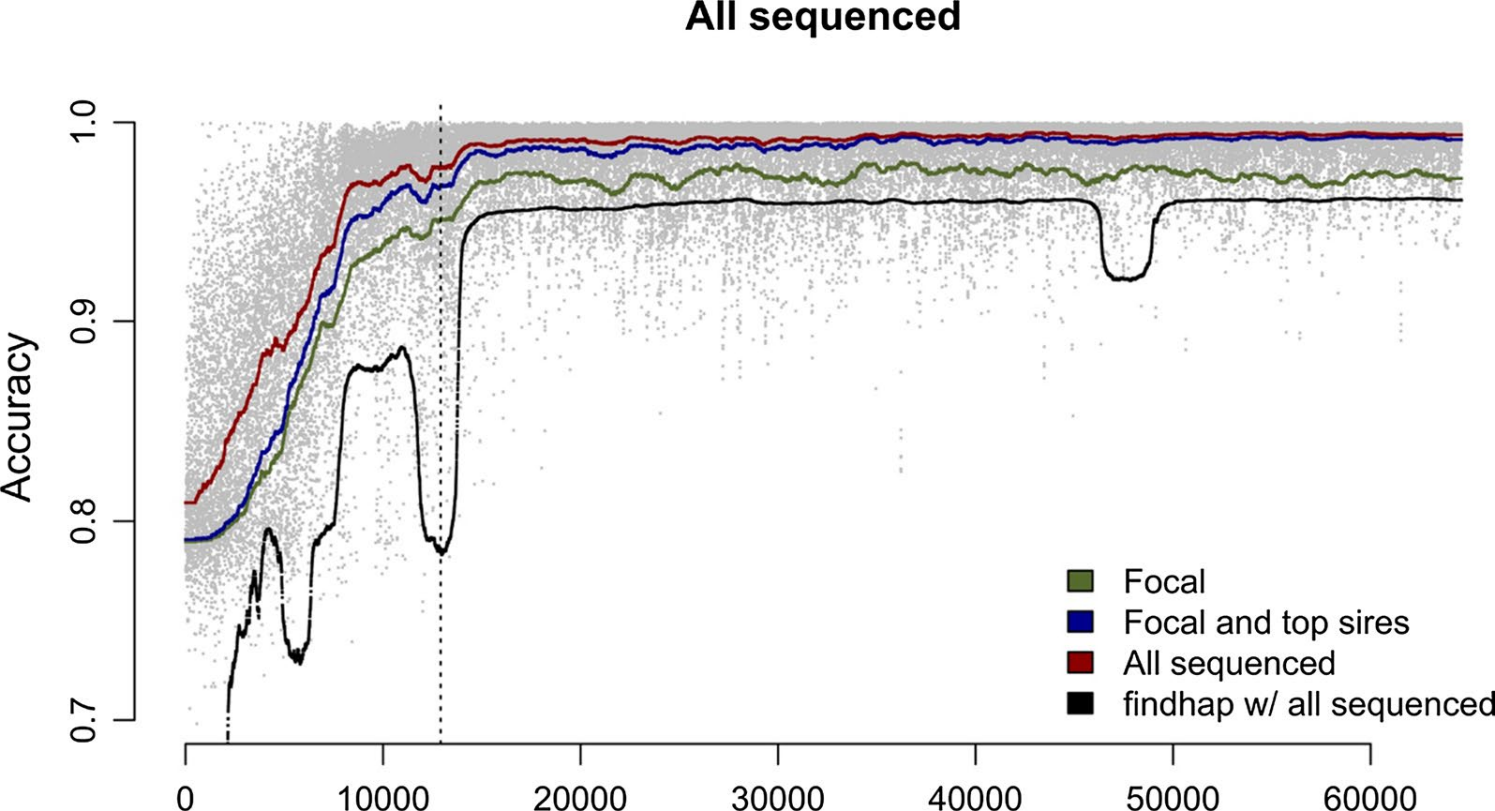
Individual imputation accuracy as a function of birth order. The lines show the rolling average for 1000 individuals when the *focal* individuals (green), the *focal and low coverage sires (blue)*, or the *focal and all low coverage (all sequenced)* individuals (red) were used for imputation with AlphaPeel. The grey dots show accuracy for each individual when the *focal and all low coverage (all sequenced)* individuals were used for imputation with AlphaPeel. The black line shows the rolling average for 1000 individuals from findhap when run with the *focal and all low coverage (all sequenced)* individuals. The vertical dotted line represents the break between the first quintile of individuals and the remaining four quintiles of individuals

Finally, we analysed the ability of hybrid peeling to impute whole-genome sequence data to all non-sequenced individuals in the pedigree. Figure 3 plots the imputation accuracy for every individual as a function of their position in their pedigree. In Table 3, we present the median imputation accuracy stratified by the sequencing sets used and the individual’s SNP array genotype status. Overall, we imputed highly accurate genotype dosages across the entire pedigree using the *focal plus all low coverage* sequencing set, with an accuracy of 0.987 for individuals with high-density SNP array data, 0.967 for individuals with low-density SNP array data, and 0.881 for non-genotyped individuals. We observed a qualitative difference in imputation accuracy in older individuals. Because of this, we stratified the results for the first quintile (first 12,919 individuals) and the entire pedigree.

**Table 3:** Median imputation accuracy for non-sequenced individuals as a function of used sequencing datasets and individual’s SNP array genotype status for (a) all non-sequenced individuals or (b) the final four quintiles of the population

	High density	Low density	No genotype
*(a) * *All individuals*			
Focal	0.967	0.936	0.855
Focal and low coverage sires	0.983	0.952	0.863
Focal and all low coverage (all sequenced)	0.987	0.971	0.881
findhap with all sequenced	0.958	0.892	0.702
*(b)* *Final four quintiles*			
Focal	0.968	0.968	0.939
Focal and low coverage sires	0.984	0.985	0.953
Focal and all low coverage (all sequenced)	0.987	0.988	0.959
findhap with all sequenced	0.959	0.916	0.765

We observed three trends in imputation accuracy. First, individuals in the first quintile had on average a lower imputation accuracy then the rest of the population. When we used the *focal plus all low coverage* sequencing set, the imputation accuracy for the first quintile was 0.908, compared to the average imputation accuracy of 0.970. This decrease in imputation accuracy is due to the lower average sequencing coverage of ancestors for individuals in the first quintile (the average coverage summed across all ancestors in the first quintile was 83x compared to the population average of 308x) and the smaller number of individuals with high-density SNP array data (0.2% in the first quintile compared to the population average of 70%).

Second, increasing the amount of sequencing resources increased accuracy for all individuals in the population. The largest contribution came from using focal individuals and their parents and grandparents, which yielded an imputation accuracy of 0.945. Furthermore, adding low coverage sequence data of top sires increased imputation accuracy to 0.963. Finally, adding sequence data of top dams and of the remaining low coverage individuals increased imputation accuracy only to 0.970, but had a proportionally larger influence on individuals in the first quintile where imputation accuracy increased from 0.885 to 0.908. This effect is likely because 78% of the top dams and top-up individuals came from the first quintile.

Third, imputation accuracy for an individual depended on its SNP array genotype status. A comparison of the imputation accuracies depending on their SNP array density is in Table 3. Overall, the difference between having high-density or low-density SNP array data tended to be small, whereas the difference between having SNP array data or not tended to be larger, although this difference decreased in the later generations. For the final four quintiles, the difference between having high-density or low-density SNP array data was negligible (both had an accuracy higher than 0.987), and the difference between having SNP array data or not was smaller than in the first quintile (0.988 vs. 0.959). In comparison, in the first quintile the difference between having high-density or low-density SNP array data was relatively larger (0.983 vs. 0.951) and the difference between having SNP array data or not was much larger (0.951 vs. 0.868).

Compared to findhap, hybrid peeling consistently yielded a higher accuracy, both in the first quintile, but also in the remaining four quintiles. Hybrid peeling outperforms findhap on individuals genotyped at high density (0.987 vs. 0.958), low density (0.967 vs. 0.892), and non-genotyped individuals (0.881 vs. 0.702). Similar to hybrid peeling, the performance of findhap was substantially lower in the first quintile, compared to the final four quintiles (mean of 0.777 vs. 0.915), but also was low in some regions, likely due to the presence of a large number of low-density or non-genotyped individuals clustered in certain portions of the pedigree.

### Computational requirements

Computational requirements were much less for hybrid peeling than for multi-locus peeling. We compared the time necessary for multi-locus peeling to process the high-density SNP array with 2000 markers used as an initial step of hybrid peeling to the time necessary for hybrid peeling to process the remaining sequence with 700,000 segregating loci when using the *focal plus all low coverage* sequencing set. We found that the initial multi-locus peeling step took 823 min and 41 GB of memory to process 2000 SNPs on 64,598 individuals, which translates to 6.3 h per 1000 individuals per 1000 loci. The hybrid peeling step was split across 1000 jobs of 700 SNPs each. Each job took on average 40 min and 2.3 GB of memory, which translates to 53.5 min per 1000 individuals per 1000 loci and a total of 40,344 min (roughly 28 core-days). The total computation time for findhap was 5.14 h and it required 101 GB of memory.

## Discussion

In this paper, we present a hybrid peeling method for calling, phasing, and imputing sequence data of any coverage in large pedigrees. This method is computationally efficient and enables the benefits of multi-locus peeling to be realised for datasets with tens of thousands of individuals on tens of millions of segregating variants. We evaluated the performance of hybrid peeling for calling and phasing sequence data in a livestock population and for imputing that sequence data to the non-sequenced individuals in the population. Hybrid peeling successfully used the pedigree to propagate information between relatives to call genotypes and phase alleles for individuals with low and high sequencing coverage. Furthermore, calling and phasing these individuals were most effective when the full pedigree was used. Hybrid peeling was also able to impute whole-genome sequence information to 65,000 animals with an accuracy higher than 0.98. We discuss these results in more detail below.

### Hybrid peeling as a genotype calling and phasing method

We found that hybrid peeling effectively used pedigree information to call genotypes and phase alleles in a population of sequenced individuals. When using hybrid peeling, sequence data from an individual’s parents and grandparents increased the yield and accuracy of called genotypes and the yield and accuracy of phased alleles compared to just using an individual’s own sequence data. We also found that further increases could be gained by using more distant relatives. The benefits of using the full pedigree were most apparent for individuals that had low coverage sequencing data (1x and 2x), for which in some cases the total genotype yield could rise from 0.1 based on the individuals own sequence data to over 0.9 using the sequence data from the entire pedigree. These results suggest that hybrid peeling could be used to increase the yield of calling and phasing sequence data in pedigrees. The application of hybrid peeling is not limited to individuals with whole-genome sequence data, but may also be useful when handling data that are generated through genotyping via a reduced-representation sequencing (e.g., RAD-seq [41] or genotyping-by-sequencing [8, 42]).

In addition to increasing genotype yield, hybrid peeling also phases many alleles. An individual’s own sequence data limits the number of its alleles that can be phased to just homozygous loci. In contrast, the number of phased heterozygous loci greatly increased with higher sequencing coverage of the individual’s parents, grandparents, or even more distant relatives. The ability to phase alleles accurately will be important for downstream imputation and other analyses. Pedigree-based methods, such as hybrid peeling offer one route for obtaining this information. There are alternative methods that are based on hidden Markov models, e.g., Beagle [17]. These methods phase an individual’s alleles by finding the shared chromosome segments between the individual and its distant relatives. However, currently these methods do not scale well for performing whole-genome sequence phasing and imputation for tens of thousands of individuals [43], making them impractical for many livestock settings.

The power of hybrid peeling comes from its ability to combine sequence data across many related individuals. Hybrid peeling identifies shared chromosome segments between parents and their progeny and propagates that information to all the individuals who share those segments. In many cases, particularly with low coverage sequence data, it is not possible to clearly identify shared chromosome segments. Hybrid peeling handles those cases by marginalizing over the possible segregation probabilities, allowing it to exploit even low coverage sequence data over many generations. When analysing the increase in performance between phasing 1x individuals in the case of disconnected families versus the case of the full pedigree, we found that the most reliable indicator of phasing accuracy was the total amount of sequencing coverage for all the individual’s ancestors, and not the amount of sequencing coverage on the individual’s parents and grandparents, suggesting that hybrid peeling is able to use even distant relatives to phase individuals.

The heavy reliance of pedigree-based imputation is both a boon and a curse for hybrid peeling. As we discuss above, using pedigree information can lead to high accuracy, high yield genotype calling and phasing for low coverage individuals. The usefulness of this technique relies on the availability of multi-generational pedigree information. Although there is some benefit in using sequence information on an individual’s parents and grandparents, the primary benefit comes from aggregating sequencing information across many generations. Multi-generational pedigree information is generally routinely available in commercial livestock populations, but may not be available for human, wild animal, and plant populations. When pedigree information is unavailable, the performance of hybrid peeling may be less than that of non-pedigree based imputation methods that rely on LD to call and phase sequence data [22]. There may be some benefit in combining linkage-based information with pedigree-based information for calling and phasing individuals in populations with shallow pedigrees where linkage information between disconnected populations can be exploited. Existing methods have already considered combining linkage-based information in the context of multi-locus peeling [33], and for using pedigree-based information in the context of linkage disequilibrium based calling and phasing algorithms [19, 44]. Future work is needed to analyse the optimal integration of hybrid peeling with linkage-based methods for use in low-depth pedigrees.

### Hybrid peeling as a whole pedigree imputation method

We found that hybrid peeling could effectively use mixed coverage sequence data to impute whole-genome sequence into the non-sequenced individuals in the pedigree. For the majority of individuals, we obtained an imputation accuracy of 0.98. Imputation accuracy was lower at the beginning than at the end of the pedigree due to the low ancestral sequencing coverage and the large number of individuals genotyped with low-density SNP arrays early in the pedigree. This trend identifies a difficulty that many pedigree-based imputation methods face, i.e., it is generally easier to impute children from their parents then it is to impute parents from their children. This difficulty arises from the fact that it is often challenging to phase parents based on their children’s genotype since it requires finding patterns of shared inheritance across multiple offspring, and generally requires many children [45]. In contrast, it is relatively easy to phase a child’s genotype based on its parents’ genotypes.

One of the more surprising results was the high accuracy observed for non-genotyped individuals. Restricted to the last four quintiles of individuals in the pedigree, non-genotyped individuals had an imputation accuracy of 0.959, which is only slightly less than the 0.988 accuracy for individuals that had high-density SNP array data. The only information that was available for hybrid peeling for non-genotyped individuals was their position in the pedigree and the list of parents, mates, and offspring. Using this information, hybrid peeling was able to accurately reconstruct inheritance of chromosomes across generations, and impute these individuals up to whole-genome sequence. The ability of hybrid peeling to impute non-genotyped pedigree members highlights the difference between pedigree- and LD-based methods such as Beagle [17], Impute2 [46], or MaCH [15], which require that all individuals are genotyped with, at least, a low-density SNP array.

We also noted significant computational gains of hybrid peeling compared to the multi-locus peeling of Meuwissen and Goddard [33]. Both methods scale linearly with the number of individuals and number of loci. However, compared to full multi-locus peeling, we found that hybrid peeling ran about 6 times faster and used less memory than full multi-locus peeling. The increased speed stems from not having to update the segregation probabilities at each locus. The decreased memory stems from being able to run each locus independently, allowing us to deallocate the memory for variables associated with the previous allele when working on the next allele. The resulting memory requirements of hybrid peeling scale linearly with the number of individuals O(N), while multi-locus peeling memory requirements scale linearly both with the number of individuals and number of loci O(NL). The gains in speed and memory also lead to practical gains in implementing hybrid peeling. Because each locus is independent of other loci given the segregation probabilities, hybrid peeling is trivial to parallelize. Furthermore, the lower memory requirement makes it possible to do this parallelization on even small machines. Parallelisation meant that although overall imputation time for 700,000 segregating loci on 64,598 individuals took 28 days of CPU time, we were able to run it on a computing cluster in under 24 h of real time.

## Conclusions

This paper presents hybrid peeling, a computationally tractable multi-locus peeling algorithm for whole-genome sequence data. We demonstrated the effectiveness of hybrid peeling in calling, phasing, and imputing whole-genome sequence in a large livestock population. We found that hybrid peeling could effectively use multiple generations of variable coverage sequence data to increase easily the yield and accuracy of called genotypes and phased alleles compared to using an individual’s own sequence data. We also found that hybrid peeling could accurately impute whole-genome sequence into non-sequenced individuals. We implemented a version of this method in the software package AlphaPeel, which is available from the AlphaGenes website (http://www.alphagenes.roslin.ed.ac.uk). Hybrid peeling has the potential to open the door to the routine utilization of whole-genome sequence in large pedigreed populations, increasing the accuracy of genomic prediction and the power to detect quantitative trait loci.

## References

[1] Meuwissen THE, Hayes BJ, Goddard ME (2001). Prediction of total genetic value using genome-wide dense marker maps. Genetics.

[2] Meuwissen T, Hayes B, Goddard M (2016). Genomic selection: a paradigm shift in animal breeding. Anim Front.

[3] Wellcome Trust Case Control Consortium (2007). Genome-wide association study of 14,000 cases of seven common diseases and 3,000 shared controls. Nature.

[4] Visscher PM, Wray NR, Zhang Q, Sklar P, McCarthy MI, Brown MA (2017). 10 Years of GWAS discovery: biology, function, and translation. Am J Hum Genet.

[5] Daetwyler HD, Villanueva B, Woolliams JA (2008). Accuracy of predicting the genetic risk of disease using a genome-wide approach. PLoS One.

[6] Hayes BJ, Visscher PM, Goddard ME (2009). Increased accuracy of artificial selection by using the realized relationship matrix. Genet Res (Camb).

[7] Hickey JM, Dreisigacker S, Crossa J, Hearne S, Babu R, Prasanna BM (2014). Evaluation of genomic selection training population designs and genotyping strategies in plant breeding programs using simulation. Crop Sci.

[8] Gorjanc G, Cleveland MA, Houston RD, Hickey JM (2015). Potential of genotyping-by-sequencing for genomic selection in livestock populations. Genet Sel Evol.

[9] Hickey JM (2013). Sequencing millions of animals for genomic selection 2.0. J Anim Breed Genet.

[10] Daetwyler HD, Capitan A, Pausch H, Stothard P, van Binsbergen R, Brøndum RF (2014). Whole-genome sequencing of 234 bulls facilitates mapping of monogenic and complex traits in cattle. Nat Genet.

[11] Veerkamp RF, Bouwman AC, Schrooten C, Calus MPL (2016). Genomic prediction using preselected DNA variants from a GWAS with whole-genome sequence data in Holstein-Friesian cattle. Genet Sel Evol.

[12] Cheung CYK, Marchani Blue E, Wijsman EM (2014). A statistical framework to guide sequencing choices in pedigrees. Am J Hum Genet.

[13] Gonen S, Ros-Freixedes R, Battagin M, Gorjanc G, Hickey JM (2017). A method for the allocation of sequencing resources in genotyped livestock populations. Genet Sel Evol.

[14] Ros-Freixedes R, Gonen S, Gorjanc G, Hickey JM (2017). A method for allocating low-coverage sequencing resources by targeting haplotypes rather than individuals. Genet Sel Evol.

[15] Li Y, Willer CJ, Ding J, Scheet P, Abecasis GR (2010). MaCH: using sequence and genotype data to estimate haplotypes and unobserved genotypes. Genet Epidemiol.

[16] Browning BL, Browning SR (2016). Genotype imputation with millions of reference samples. Am J Hum Genet.

[17] Browning SR, Browning BL (2007). Rapid and accurate haplotype phasing and missing-data inference for whole-genome association studies by use of localized haplotype clustering. Am J Hum Genet.

[18] Browning BL, Browning SR (2009). A unified approach to genotype imputation and haplotype-phase inference for large data sets of trios and unrelated individuals. Am J Hum Genet.

[19] O’Connell J, Gurdasani D, Delaneau O, Pirastu N, Ulivi S, Cocca M (2014). A general approach for haplotype phasing across the full spectrum of relatedness. PLoS Genet.

[20] Hickey JM, Kinghorn BP, Tier B, Wilson JF, Dunstan N, van der Werf JH (2011). A combined long-range phasing and long haplotype imputation method to impute phase for SNP genotypes. Genet Sel Evol.

[21] Cheung CYK, Thompson EA, Wijsman EM (2013). GIGI: an approach to effective imputation of dense genotypes on large pedigrees. Am J Hum Genet.

[22] VanRaden PM, Sun C, O’Connell JR (2015). Fast imputation using medium or low-coverage sequence data. BMC Genet.

[23] Elston RC, Stewart J (1971). A general model for the genetic analysis of pedigree data. Hum Hered.

[24] Cannings C, Thompson EA, Skolnick HH (1976). The recursive derivation of likelihoods on complex pedigrees. Adv Appl Probab.

[25] Cannings C, Thompson EA, Skolnick MH (1978). Probability functions on complex pedigrees. Adv Appl Probab.

[26] Lander ES, Green P (1987). Construction of multilocus genetic linkage maps in humans. Proc Natl Acad Sci USA.

[27] Fernández SA, Fernando RL, Guldbrandtsen B, Totir LR, Carriquiry AL (2001). Sampling genotypes in large pedigrees with loops. Genet Sel Evol.

[28] Totir LR, Fernando RL, Abraham J (2009). An efficient algorithm to compute marginal posterior genotype probabilities for every member of a pedigree with loops. Genet Sel Evol.

[29] Lauritzen SL, Sheehan NA (2003). Graphical models for genetic analyses. Stat Sci.

[30] Bishop CM (2007). Pattern recognition and machine learning.

[31] Koller D, Friedman N (2009). Probabilistic graphical models: principles and techniques.

[32] Kerr RJ, Kinghorn BP (1996). An efficient algorithm for segregation analysis in large populations. J Anim Breed Genet.

[33] Meuwissen T, Goddard M (2010). The use of family relationships and linkage disequilibrium to impute phase and missing genotypes in up to whole-genome sequence density genotypic data. Genetics.

[34] Piccolboni A, Gusfield D (2003). On the complexity of fundamental computational problems in pedigree analysis. J Comput Biol J.

[35] Van Arendonk JAM, Smith C, Kennedy BW (1989). Method to estimate genotype probabilities at individual loci in farm livestock. Theor Appl Genet.

[36] Rabiner L (1989). A tutorial on hidden Markov models and selected applications in speech recognition. Proc IEEE.

[37] Chen GK, Marjoram P, Wall JD (2009). Fast and flexible simulation of DNA sequence data. Genome Res.

[38] Faux A-M, Gorjanc G, Gaynor RC, Battagin M, Edwards SM, Wilson DL (2016). AlphaSim: software for breeding program simulation. Plant Genome.

[39] MacLeod IM, Larkin DM, Lewin HA, Hayes BJ, Goddard ME (2013). Inferring demography from runs of homozygosity in whole-genome sequence, with correction for sequence errors. Mol Biol Evol.

[40] Calus MPL, Bouwman AC, Hickey JM, Veerkamp RF, Mulder HA (2014). Evaluation of measures of correctness of genotype imputation in the context of genomic prediction: a review of livestock applications. Animal.

[41] Davey JW, Hohenlohe PA, Etter PD, Boone JQ, Catchen JM, Blaxter ML (2011). Genome-wide genetic marker discovery and genotyping using next-generation sequencing. Nat Rev Genet.

[42] Elshire RJ, Glaubitz JC, Sun Q, Poland JA, Kawamoto K, Buckler ES (2011). A robust, simple genotyping-by-sequencing (GBS) approach for high diversity species. PLoS One.

[43] Gilly A, Kuchenbaecker K, Southam L, Suveges D, Moore R, Melloni G, et al. Very low depth whole genome sequencing in complex trait association studies. bioRxiv [Internet]. 2017. http://biorxiv.org/content/early/2017/07/28/169789.abstract. 10.1093/bioinformatics/bty1032 PMC6662288 30576415

[44] Chen W, Li B, Zeng Z, Sanna S, Sidore C, Busonero F (2013). Genotype calling and haplotyping in parent-offspring trios. Genome Res.

[45] Ferdosi MH, Kinghorn BP, van der Werf JHJ, Gondro C (2014). Detection of recombination events, haplotype reconstruction and imputation of sires using half-sib SNP genotypes. Genet Sel Evol.

[46] Howie BN, Donnelly P, Marchini J (2009). A flexible and accurate genotype imputation method for the next generation of genome-wide association studies. PLoS Genet.

